# Adeno-Associated Viral Vector-Mediated Transgene Expression Is Independent of DNA Methylation in Primate Liver and Skeletal Muscle

**DOI:** 10.1371/journal.pone.0020881

**Published:** 2011-06-08

**Authors:** Adrien Léger, Caroline Le Guiner, Michael L. Nickerson, Kate McGee Im, Nicolas Ferry, Philippe Moullier, Richard O. Snyder, Magalie Penaud-Budloo

**Affiliations:** 1 INSERM UMR649, Nantes, France; 2 Généthon, Evry, France; 3 National Cancer Institute, National Institutes of Health, Frederick, Maryland, United States of America; 4 INSERM UMR948, Nantes, France; 5 Department of Molecular Genetics and Microbiology, College of Medicine, University of Florida, Gainesville, Florida, United States of America; 6 Center of Excellence for Regenerative Health Biotechnology, University of Florida, Alachua, Florida, United States of America; University of Kansas Medical Center, United States of America

## Abstract

Recombinant adeno-associated viral (rAAV) vectors can support long-term transgene expression in quiescent tissues. Intramuscular (IM) administration of a single-stranded AAV vector (ssAAV) in the nonhuman primate (NHP) results in a peak protein level at 2–3 months, followed by a decrease over several months before reaching a steady-state. To investigate transgene expression and vector genome persistence, we previously demonstrated that rAAV vector genomes associate with histones and form a chromatin structure in NHP skeletal muscle more than one year after injection. In the mammalian nucleus, chromatin remodeling *via* epigenetic modifications plays key role in transcriptional regulation. Among those, CpG hyper-methylation of promoters is a known hallmark of gene silencing. To assess the involvement of DNA methylation on the transgene expression, we injected NHP *via* the IM or the intravenous (IV) route with a recombinant ssAAV2/1 vector. The expression cassette contains the transgene under the transcriptional control of the constitutive Rous Sarcoma Virus promoter (RSVp). Total DNA isolated from NHP muscle and liver biopsies from 1 to 37 months post-injection was treated with sodium bisulfite and subsequently analyzed by pyrosequencing. No significant CpG methylation of the RSVp was found in rAAV virions or in vector DNA isolated from NHP transduced tissues. Direct *de novo* DNA methylation appears not to be involved in repressing transgene expression in NHP after gene transfer mediated by ssAAV vectors. The study presented here examines host/vector interactions and the impact on transgene expression in a clinically relevant model.

## Introduction

Recombinant Adeno-Associated Virus (rAAV)-mediated gene transfer can sustain long-term expression of a protein in large animal models after a single administration in skeletal muscle [1]–[4]. After intramuscular (IM) injection of single-stranded AAV (ssAAV) vectors in nonhuman primates (NHP), a rise in the level of vector-expressed secreted proteins is seen in the serum during the first 2–3 months (Phase 1), followed by a progressive decrease (Phase 2) [1]–[4]. The protein concentration reaches a steady-state (Phase 3) 6–8 months post-injection (pi) at half the maximum level. After IM injection of ssAAV vectors in mouse, the maximal protein expression is also attained in ≈10 weeks. Phase 2 reduction seems however less obvious [5]–[7]. Whereas rAAV trafficking and double-stranded conversion characterizing Phase 1 were extensively studied [8], [9], the mechanism(s) underlying decreasing protein level in Phase 2 in NHP has not been elucidated.

Recombinant AAV vector genomes persist mainly as circular episomes (monomers and high-molecular weight concatemers) in NHP muscle and liver [4], [10], [11]. These molecular structures seem to be more heterogeneous in macaque than in murine liver [10]. Similar to other non-integrating viruses [12], [13], we recently demonstrated that the rAAV episomes assimilate, in NHP skeletal muscle, into a chromatin-like structure where nucleosomes are regularly assembled along the viral genome in a pattern similar to cellular chromatin [4]. Chromatinization may contribute to the stability of rAAV DNA in quiescent tissues, but could also lead to epigenetic-mediated modulation of transgene expression. Indeed, in mammals, chromatin structure accessibility is affected by two main epigenetic modifications: post-transcriptional modifications (PTM) of histones [14] and direct DNA methylation on cytosine of the CG dinucleotides (CpG) [15], [16]. These modifications act in cooperation to influence transcription levels in a positive or a negative manner [17].

In mammals, CpG hypermethylation of promoters is known to be associated with inactivation of gene expression [15], [16]. With regards to gene therapy, CpG methylation has been identified as a mark of transgene silencing after MLV retroviral vector transduction [18], [19]. DNA methylation was also considered as a potential cause of transcriptional repression after plasmid-mediated gene expression [20], [21]. Nevertheless, it seems that histone modifications are more involved in this regulation than DNA methylation *per se* [22], [23]. Concerning wild-type episomal viruses, the situation is variable. DNA methylation plays a crucial role in the Epstein Barr Virus (EBV) lifecycle for example, but is not involved in gene regulation of other herpes viruses [24]. We therefore investigated whether rAAV vector genomes are subject to DNA methylation and partially silenced after gene transfer in skeletal muscle and liver of a clinically relevant model, the NHP.

Next generation sequencing technologies were recently shown to be powerful and reproducible methods to quantify CpG methylation [25]–[28]. Thus, we performed pyrosequencing analyses after sodium bisulfite conversion of vector molecules that were isolated directly from rAAV-transduced NHP tissues. We quantified the methylation percentage on each CpG of the constitutive Rous Sarcoma Virus promoter (RSVp). The RSVp contains a CpG region of intermediate density [29], [30], and this category of promoter has been reported as most sensitive to *de novo* DNA methylation [31]. Indeed, several studies have shown that even low levels of methylation in the RSV long terminal repeat (LTR) can be sufficient for its transcriptional inactivation [32]–[34]. In the present study, we established that the CpGs located in the RSVp are unmethylated in the rAAV vector virion stocks, and that rAAV genomes are not subject to *de novo* methylation in NHP skeletal muscle and liver at early (1 month) to late time points (up to 37 months). In order to directly correlate the local transgene expression with the lack of rAAV vector DNA methylation, a quantitative RT-PCR assay was validated and a dramatically higher transgenic RNA level was seen in the skeletal muscle compared to the liver from the same animals. Thus, the majority of rAAV-derived transgene protein is expressed from the transduced skeletal muscle. After analyzing the rAAV genome population and individual rAAV genomes isolated from transduced muscle, our data show that DNA methylation is not involved in the partial suppression of transgene expression after ssAAV vector administration in the NHP.

## Materials and Methods

### Ethics Statement

Experiments were conducted on 3–5 kg captive-bred cynomolgus macaques purchased from BioPrim (Baziège, France). Macaques were housed in an enriched environment (toys, fresh fruits and vegetables) and were monitored daily for health and welfare. The Institutional Animal Care and Use Committee of the Pays de Loire (France) approved the protocol (permit number #2006.5). The research was conducted at the Boisbonne Centre (ONIRIS, Nantes) under the authorizations #5937C and #005608D delivered by the Departmental Direction of Veterinary Services (Loire-Atlantique, France) and in accordance with the recommendations of the Weatherall report: “The use of nonhuman primates in research”.

The experimental protocol was designed in a previous study [2]. In order to avoid any discomfort during and after the experiments, all procedures were carried out after animal sedation with 30 µg/kg of Medetomidine (Domitor®, Pfizer) and 7 mg/kg of Ketamine (Imalgene®, Merial). Intramuscular and intravenous injections of rAAV vectors were classified as mild severity procedures and skeletal muscle and liver biopsies as moderate level severity. Surgeries were performed under Isofluorane anesthesia, monitoring clinical parameters (temperature, blood pressure, heart rate, respiratory rate…). Pre-operative analgesia was obtained by intra-cutaneous injection of 0.1 mg/kg of Morphine and a treatment with a non-steroidal anti-inflammatory drug (0.1 mg/kg of Meloxicam, Metacam®) was orally administered after surgery for 3 days. Special attention was paid to the health and welfare of animals during the work, and blood samples were collected regularly to follow biochemical and hematological parameters.

Mouse experiments were conducted under the agreement #A44-124 delivered by the Departmental Direction of Veterinary Services (Loire-Atlantique, France) and in accordance with the French law concerning experimentations on vertebrate laboratory animals (Décret 87-848, 1987). All animals were handled in accordance with the Guide for the Care and Use of Laboratory Animals.

### Recombinant AAV vector production and *in vivo* administration

The pZA-RSV-LEA29Y-WPRE-pA vector plasmid and the rAAV2/1 vector virions production were described previously by Toromanoff *et al.* [2]. The expression cassette is composed of the Rous Sarcoma Virus (RSV) promoter that drives the expression of the immunosuppressive LEA29Y (belatacept®) protein. Five cynomolgus macaques were injected IM in the tibialis anterior muscle (Mac 1, Mac 2, and Mac 9) [2] or IV in the right external saphenous vein (Mac 10 and Mac 11) with rAAV2/1 at a dose of 5.10^12^ vector genomes (vg) per kg.

The pAAV-RSV-GFP-pA vector plasmid contains the green fluorescent protein (GFP) sequence between the RSVp and the SV40 polyA signal sequences. Ten week-old C57B/6J mice were injected in the two tibialis anterior muscles with a total dose of 2.8.10^12^ vg/kg of single-stranded AAV2/8-RSV-GFP-pA vector. Animals were sacrificed at 7 days post-injection.

### Total DNA and viral DNA extraction

Total DNA was extracted by incubating minced NHP skeletal muscle or liver biopsies in urea buffer and proteinase K at 56°C and subsequently purified by a phenol-chloroform organic extraction. Recombinant AAV viral DNA was recovered using NucleoSpin® Blood Kit (Macherey Nagel) from 2.10^12^ vg of rAAV2/1-RSV-LEA29Y-WPRE per column according to the manufacturer instructions.

### Quantitative PCR

Primers and TaqMan probes used for the amplification of vector-specific sequence (LEA29Y) and of endogenous macaque sequence (macaque ε-globin gene), as well as Q-PCR conditions were previously described by Toromanoff *et al.* [2].

### RNA extraction and quantitative RT-PCR

RNA was isolated from NHP tissues by mechanical shredding followed by organic extraction using TRI Reagent® (Ambion). Residual DNA was removed from RNA preparations using the TURBO DNA-*free*™ kit (Ambion). The reverse transcription was performed using the *M*-*MLV*-reverse transcriptase (Invitrogen). We quantified the transgene-derived mRNA by targeting the WPRE sequence using primers and Q-PCR conditions described by Lizée *et al.* [35] for NHP samples and using the following primers for murine samples: GFP-A 5′-ACTACAACAGCCACAACGTCTATATCA and GFP-B 5′-GGCGGATCTTGAAGTTCACC. We also performed control Q-RT-PCR on the Myosin-4 (MYH4) target gene expressed in the muscle but not in the liver, and on the Albumin (ALB1) gene displaying the opposite pattern. The following primers and probes were used for the MYH4 mRNA quantification: forward primer 5′-GAGGTTGCTCATCGGTTT, reverse primer 5′-ATGGACACTGCTGAAGATAC, probe 5′-FAM-GGCTTGGTGCTGGTTGCT-TAMRA and for the ALB1 mRNA quantification: forward primer 5′-GAGGTTGCTCATCGGTTT, reverse primer 5′-ATGGACACTGCTGAAGATAC, probe 5′-FAM-GGCTTGGTGCTGGTTGCT-TAMRA. All Q-RT-PCR were conducted using an ABI StepOne Plus machine (Applied Biosystems) with TaqMan chemistry, at the exception of GFP and mouse HPRT mRNA amplification that were performed using SYBR Green chemistry. The TaqMan PCR were done using the following program: initial denaturation at 95°C for 20 s followed by 45 cycles of 3 s at 95°C and 30 s at 62°C. The SYBR Green amplifications were performed as followed: initial denaturation at 95°C for 20 s followed by 40 cycles of 3 s at 95°C and 30 s at 60°C. Q-RT-PCR data were normalized to the stably expressed HPRT mRNA for NHP samples [36], [37]. For murine samples, 11 potential reference genes were evaluated using the Mouse Endogenous Control Gene Panel kit (Tataa biocenter). The HPRT reference gene was determined after Genex standard software (Tataa biocenter) analysis as to be the most constantly expressed gene in mouse muscle and liver. Thus, for both species, the Ct results obtained for the transgene were normalized to the HPRT mRNA corresponding values using the equation RQ = 2^−(Ct target – Ct reference)^.

### Generation of unmethylated and fully methylated controls

The unmethylated version of the RSVp sequence was generated by PCR amplification of the pZA-RSV-LEA29Y-WPRE-pA vector plasmid using the pRSV F/pRSV R primer set (Table 1). The fully methylated version of the RSVp was generated from the previous control by *in vitro* methylation using M.Sss1 CpG methyltransferase (New England Biolabs) according to the manufacturer's instructions for high density CpG DNA.

**Table 1:** PCR primers used for RSVp methylation analysis.

Primers	Primer sequence	Target	Amplicon size (bp)
pRSV F	5′-GTGTTGGAGGTCGCTGAGTAGT	RSVp	749
pRSV R	5′-CAGACTGAGCAGCGTCCTCT		
pRSV Pyro1F	5′-TGTATGAAGAATTTGTTTAGGGTTAG	Pyro1 after bisulfite conversion	188
pRSV Pyro1R Biot	5′-[Btn]-ATAAAACTACATTTCCCCCTCC		
pRSV Pyro2F	5′-TGTTTTATAAGGAGAGAAAAAGTAT	Pyro2 after bisulfite conversion	224
pRSV Pyro2R Biot	5′-[Btn]-TGTTTTATAAGGAGAGAAAAAGTAT		

### Sodium bisulfite conversion and low-throughput pyrosequencing (LTS)

The optimal amount of 500 ng of total DNA extracted from transduced NHP tissues was submitted to a sodium bisulfite treatment using the EZ DNA Methylation-Gold kit (Zymo research). In parallel, 10 ng of unmethylated or fully methylated control were mixed with 500 ng of total DNA extracted from naïve NHP skeletal muscle or liver and submitted to the same treatment. The Pyro1 and Pyro2 CpG-rich regions of the RSVp were then amplified by PCR using the following program: 5 min at 95°C followed by 50 cycles of 20 s at 95°C, 30 s at 58°C (Pyro1) or 52°C (Pyro2) and 15 s at 72°C. Each reaction was performed in a final volume of 50 µl containing 10 ng of bisulfite converted DNA, 2.5 U of Amplitaq Gold DNA polymerase (Applied Biosystems), 5 µl of 10× Amplitaq buffer, 0.2 µM of the forward primer, 0.2 µM of the 5′biotinylated reverse primer (Table 1), 800 µM of dNTP mix and 4 mM (Pyro1) or 8 mM (Pyro2) of MgCl_2_. PCR products were purified with streptavidin coated Sepharose HP beads (Amersham Biosciences) and the Vacuum Prep Tool (Qiagen). Pyrosequencing was performed on the PSQ96MA instrument (Qiagen) using PyroMark Gold Q96 Reagents (Qiagen). Sequencing primers were designed on the upper strand of the rAAV and are listed in Table 2. Data were acquired and analyzed with PyroQ-CpG software 2.0 (Qiagen). Analysis was considered as “passed” if the non-CpG cytosines internal controls were completely converted in T.

**Table 2:** Sequencing primers used for low-throughput sequencing.

Primers	Primer sequence	Target
Pyro1 Seq A	5′-GAAGAATTTGTTTAGGGTT	Pyro1 after bisulfite conversion
Pyro1 Seq B	5′-GGGGATTAGGGTGTG	
Pyro2 Seq A	5′-TTTTATAAGGAGAGAAAAAG	Pyro2 after bisulfite conversion
Pyro2 Seq B	5′-GGTTTGATATGGATTGGA	

### 454 high-throughput sequencing (HTS)

Sodium bisulfite converted products were amplified by a two-step methylation-independent PCR reaction in order to introduce sample specific adapters used for the HTS workflow reaction (454/Roche, Branford, CT). The first PCR step included 30 cycles of amplification with the conventional primers (Table 1) that were used previously to amplify Pyro1 and Pyro2 regions after bisulfite conversion. The second step incorporated HTS primers (Table S1) to create Titanium amplicon libraries. The composite HTS primers contained the 454 primer A (forward) or B (reverse) sequence, a MID (multiplex identifier) bar code which allowed sample multiplexing, and the previously used LTS primer sequence (Table 1). The annealing temperature was increased after each cycle by +2°C until it reached the fusion primer Tm (66°C). PCR products were examined by agarose gel electrophoresis to ensure product amplification of the expected size with no background and were quantitated using an Agilent 2100 Bioanalyzer (Santa Clara, CA). Barcoded samples were pooled and run in duplicate in separate regions of a picotiter plate using Titanium reagents on a Genome Sequencer FLX system (Roche, Indianapolis, IN) according to standard procedures (Roche/454 Technical Bulletin TCB 005-2009). Sequencing reads containing individual barcodes were identified and sorted using the Amplicon Variant Analyzer Software (454, Branford, CT). The Genome Sequencer Reference Mapper (gsMapper, Roche) program was used to align reads to the reference sequences and was run using default parameters except that all reads were treated individually. “High confidence” data were extracted using the following criteria: the difference regarding the RSVp theoretical sequence was observed in forward and reverse reads and the read length was at least 135 and 160 bp for Pyro1 and 2, respectively. Finally, individual CpG were viewed in alignments of HTS reads using the Integrated Genomics Viewer (http://www.broadinstitute.org/igv/, Broad Institute, Cambridge, MA).

### Statistical analysis of pyrosequencing results (LTS and HTS)

Since we had to compare values on 22 CpGs, we used a non-parametric statistical hypothesis test: the Mann-Withney U Test. We considered that methylation level was significant above the sensitivity threshold determined using unmethylated control, if p value is under 0.001. Analyses were performed using GraphPad PRISM 5.

## Results

### RSV promoter in the rAAV virions is not methylated

**Figure 1:**
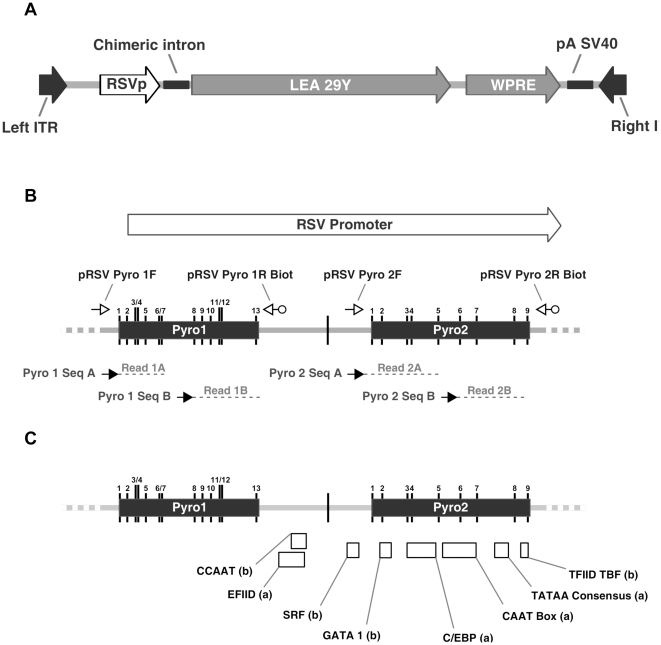
Pyrosequencing assay for the Rous Sarcoma Virus promoter (RSVp) and transcription factor binding sites. (A) Schematic representation of the rAAV-RSV-LEA29Y-WPRE-pA genome. The LEA29Y reporter gene sequence was cloned upstream of the Rous sarcoma virus (RSV) promoter and downstream of the Woodchuck hepatitis virus posttranscriptional regulatory element (WPRE). (B) Two high-density CpGs plots were identified in the RSVp, named Pyro1 and Pyro2 (black boxes). CpG positions are marked by black vertical lines and numbered from 1 to 13 for the 1^st^ CpG-rich region, Pyro1, and from 1 to 9 to the 2^nd^ region, Pyro2. PCR primers used to specifically amplify bisulfite-converted rAAV vector DNA are represented as small open arrows. Reverse primers are 5′-biotinylated allowing specific capture and subsequent purification of the PCR product. Pyrosequencing allowed examination of 22 of 23 CpGs from the RSVp using the 4 sequencing primers represented as small black arrows. (C) Transcription factor binding sites positions along the RSVp. (a) TF binding sites described by Mobley *et al.* [53]. (b) TF binding sites predicted by *in silico* analysis using EMBOSS TFscan (http://mobyle.pasteur.fr).

The rAAV vector expression cassette used here encodes the LEA29Y molecule under the control of the RSVp (Fig. 1a). We focused our investigation on the RSV promoter since CpG hyper-methylation of promoters has been shown to be critical for repression of transcription [15]. DNA methylation analyses were conducted after sodium bisulfite treatment of DNA samples. The bisulfite treatment and the subsequent RSVp-targeting PCR step convert unmethylated C into T, whereas the methylated C remains unmodified. Finally, the methylation percentage on each CpG in the population was measured by low-throughput pyrosequencing (LTS) based on the C/T ratio quantification. Two CpG-rich regions named Pyro1 and Pyro2 were identified in the RSVp by EMBOSS CpG plot (Fig. 1b) and two primers sets were designed to analyze 22 of the 23 CpGs of these two regions (Fig. 1b).

**Figure 2:**
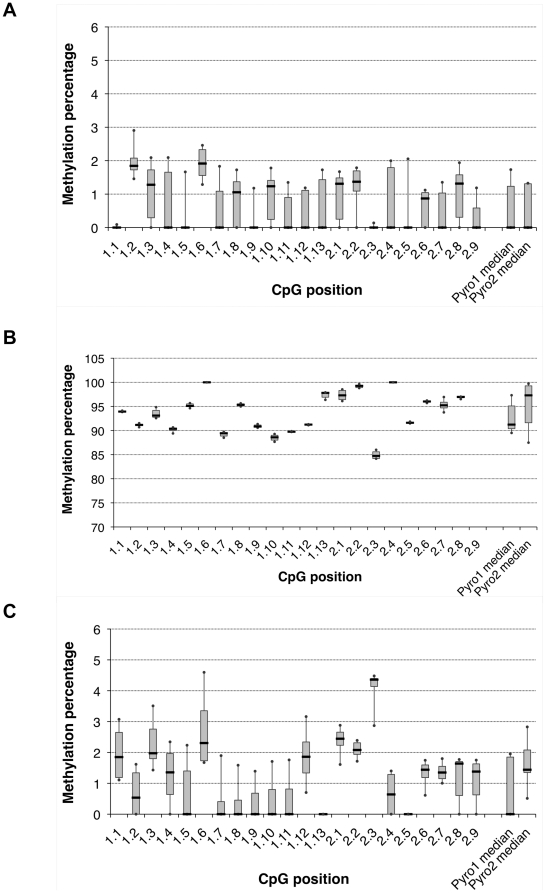
Pyrosequencing assay sensitivity and methylation status of rAAV DNA in virions. Samples were sequenced a minimum of 5 times by low-throughput sequencing and are represented on a box plot graph. CpG methylation percentages are displayed depending on the CpG position: 1.1 to 1.13 for Pyro1 region and 2.1 to 2.9 for Pyro2 region of the RSVp. Median values are represented by black horizontal lines. Grey boxes contain 50% of values and extreme values are delimited as error bars. (A) Unmethylated control generated by PCR amplification of the RSVp sequence from the pZA-RSV-LEA29Y-WPRE-pA plasmid. (B) Methylated control obtained by M.Sss1 in vitro methylation of the unmethylated RSVp amplicon. (C) Viral DNA in particles. Viral DNA was extracted from rAAV2/1-RSV-LEA29Y-WPRE particles.

The LTS assay was validated by generating an unmethylated RSVp amplicon by PCR and a fully methylated fragment by subsequent M.Sss1 *in vitro* methylation. Both controls were spiked into NHP gDNA and the bisulfite-treated products were amplified by PCR with the same efficiency, indicating that no amplification bias existed in favor of one of the two targets (data not shown). The CpG methylation median was determined by LTS for the unmetylated control and was equal to 0.00% (ΔQ1-0.00; ΔQ3+1.21) and 0.00% (ΔQ1-0.00; ΔQ3+1.24) for Pyro1 and Pyro2 plots, respectively (Fig. 2a). The sensitivity threshold of the assay is 1.87% since 90% of the values obtained for Pyro1 and Pyro2 were lower. The medians obtained for the fully methylated control were 91.23% (−0.85; +3.87) and 97.30% (−5.69; +1.97) for Pyro1 and Pyro2 regions (Fig. 2b).

In order to assess the potential *de novo* DNA methylation after vector administration, we measured the baseline methylation status of the RSVp in the purified rAAV vector virions produced in 293 cells [38]. Viral DNA was extracted from particles and treated with bisulfite prior to LTS. CpG methylation medians were equal to 0.00% (−0.00; +1.85) on Pyro1 and 1.44% (−0.09; +0.64) on Pyro2 (Fig. 2c). Since these values were below the pyrosequencing sensitivity threshold, we concluded that vector DNA present in the virions prior *in vivo* administration was unmethylated.

### The RSVp of rAAV genomes extracted from NHP skeletal muscle and liver remains essentially unmethylated

To investigate the methylation status of the rAAV genome *in vivo*, five NHP were injected intramuscularly (IM) (Mac 1, 2 and 9) or intravenously (IV) (Mac 10 and 11) with the ssAAV2/1-RSV-LEA29Y-WPRE-pA vector. Each animal received a dose of 5.10^12^ vg/kg. The LEA29Y molecule was detected in the serum of the animals at different time points as previously published in Toromanoff *et al.* [2] for Mac 1 and Mac 2, and in Penaud-Budloo *et al.* [4] for Mac 9. These animals displayed a 1.5 to 2-fold reduction in protein level from ≈3 to 6 months pi, before expression reached steady state. The LEA29Y concentration in the serum of the IV-injected animals (Mac 10 and 11) was below the sensitivity threshold of the ELISA assay (data not shown).

**Figure 3:**
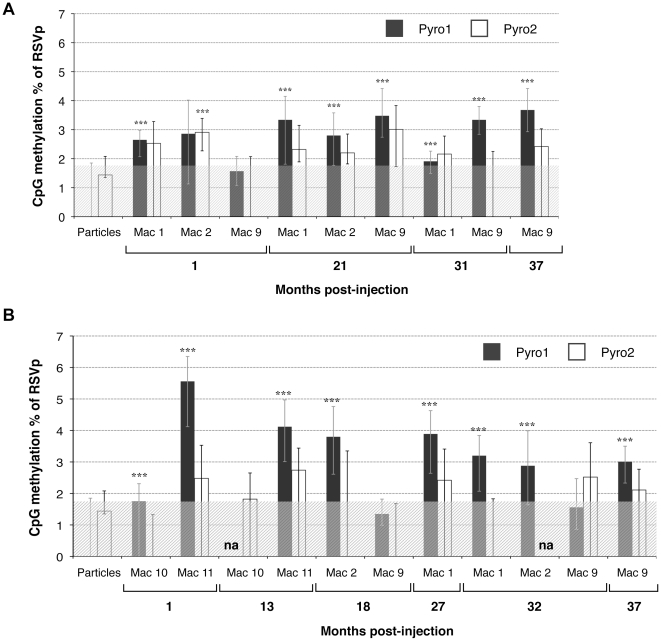
CpG methylation percentage of the RSVp after rAAV administration in NHP determined by low-throughput sequencing. NHP were injected IM (Mac 1, Mac 2, and Mac 9) or IV (Mac 10 and Mac 11) with the rAAV2/1-RSV-LEA29Y-WPRE-pA vector. Each animal received a dose of 5.10^12^ vg/kg. Total DNA was extracted from transduced skeletal muscle (A) and liver (B) and subjected to sodium bisulfite conversion and subsequent PCR amplification. Each sample was read at least 4 times by PSQ96 pyrosequencing for the two CpG-rich plots Pyro1 and Pyro2. na: not analyzable. (^***^) p value<0.001.

In order to assess the potential involvement of *de novo* DNA methylation in repression of transgene expression, total DNA was extracted from skeletal muscle and liver biopsies taken 1 to 37 months post-injection (pi) and analyzed by bisulfite LTS [26]. Independent of the time or the vector delivery route, the RSVp remains unmethylated across the Pyro2 CpG-rich region both in the skeletal muscle (Fig. 3a) and in the liver (Fig. 3b). On the contrary, most of the samples exhibited a low (below 6%) but significant methylation level on the Pyro1 plot (Fig. 3). No particular CpG site in the Pyro1 sequence was observed as being preferentially methylated (data not shown).

**Figure 4:**
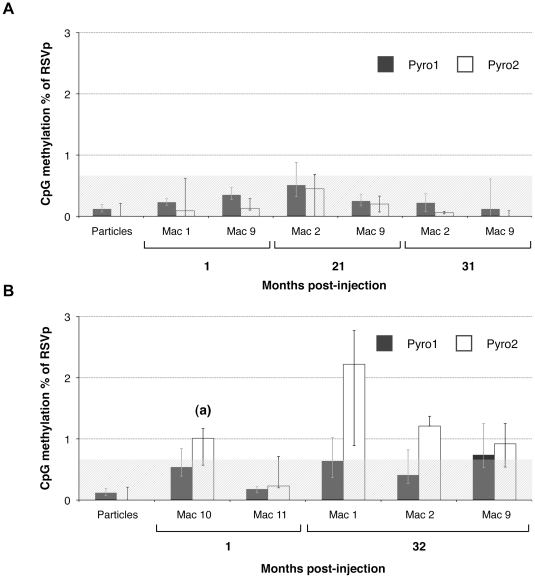
CpG methylation percentage of the RSVp after rAAV administration in NHP determined by high-throughput sequencing. NHP were injected IM (Mac 1, Mac 2, and Mac 9) or IV (Mac 10 and Mac 11) with the rAAV2/1-RSV-LEA29Y-WPRE-pA vector. Each animal received a dose of 5.10^12^ vg/kg. Total DNA was extracted from transduced skeletal muscle (A) and liver (B) and subjected to a sodium bisulfite conversion and subsequent PCR amplification. 454 sequencing was performed to evaluate the Pyro1- and Pyro2-region methylation. (a) Bioinformatic analysis of this sample revealed some sequences for which all the CpGs were methylated. (^***^) p value<0.001.

LTS technology provides a global assessment corresponding to the overall degree of methylation of the population of rAAV molecules. However, a small fraction of molecules could potentially be highly methylated but may remain undetectable when analyzing the population. In order to study individual rAAV genomic sequences, high-throughput sequencing (HTS) [39] that generated clonal sequences from individual rAAV molecules was performed on the same primate samples. PCR primers (Table S1) were designed to amplify the two main CpG-rich regions and were composed of a 454 sequencing primer, a unique barcode tag also called multiplex identifier (MID) which allowed sample multiplexing, and the pRSV Pyro1F, 1R, 2F or 2R primer sequence previously used in the LTS assay (Fig. 1b). This HTS approach created clonal sequencing reads that could be individually scored and counted. Analysis of the unmethylated control by HTS resulted in Pyro1 and Pyro2 methylation percentages of 0.37% (ΔQ1-0.15; ΔQ3+0.08) and 0.30% (ΔQ1-0.05; ΔQ3+0.31), respectively. 90% of the values were below 0.60% of methylation, hence, the HTS assay sensitivity threshold was lower than for the LTS. Results obtained for the fully methylated control were 93.31% (−3.74; +2.54) and 98.31% (−0.72; +0.73) for Pyro1 and Pyro2, respectively. Vector genomes extracted from rAAV particles gave a value of 0.12% (−0.05; +0.07) methylation for Pyro1 and 0.00% (−0.00; +0.21) for Pyro2 (Fig. 4) confirming the unmethylated nature of the RSVp in rAAV virions.

Muscle and liver NHP samples were processed with an anticipated average of 2000 clonal sequencing reads per sample. The average yield obtained for Pyro1 and Pyro2 was 7720 reads (from 809 to 12414) and 1000 reads (from 221 to 3138), respectively. The yield variation observed between Pyro1 and Pyro2 could be explained, in part, by differences in the amplicon length [28] (Table 1). Despite the high depth of sequencing read coverage, no significant DNA methylation was detected by HTS at the 22 CpG positions of the RSVp either in the NHP skeletal muscle (Fig. 4a) or in the liver (Fig. 4b) at early or late time points. Compared to LTS, smaller standard deviations were observed indicating a higher degree of reproducibility. Bioinformatic analyses of individual sequences confirmed that rAAV molecules were not methylated on the RSVp, except for Mac10 at 1 month pi in the liver (Fig. 4b) for which we detected 0.41% of reads (3130 depth of coverage) methylated on all the CpGs of the Pyro2 region. Such rare events could not be detected by LTS confirming the usefulness of the complementary HTS approach. In conclusion, we demonstrated that the RSVp was not subjected to *de novo* DNA methylation after rAAV transduction of NHP muscle and liver, up to 3 years following vector administration.

### Single-stranded rAAV-mediated gene expression is minor in the liver compared to NHP skeletal muscle

**Figure 5:**
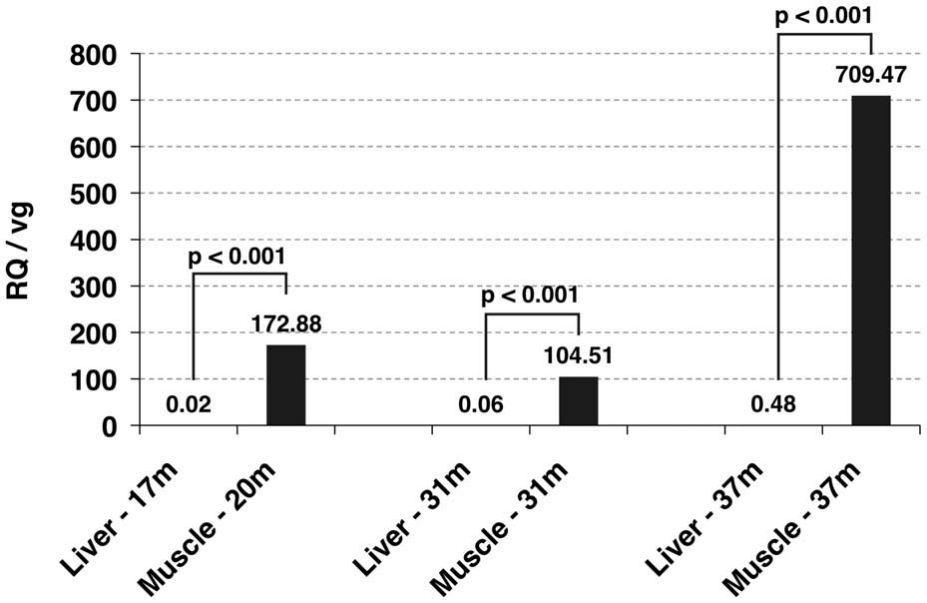
Differential transgene expression level in Mac 9 skeletal muscle and liver. mRNA relative quantities (RQ) were determined at the indicated time points by quantitative RT-PCR using WPRE as target rAAV sequence and HPRT as endogenous macaque reference, and the equation relating these: RQ = 2^−(Ct WPRE – Ct HPRT)^. Data are expressed relative to the vector genome copy number (vg) determined by Q-PCR using LEA29Y as target and ε-globin macaque gene as endogenous.

We have previously shown that the rAAV-mediated expression of the LEA29Y transgene under the RSVp transcriptional control was stable for more than 2 years in IM injected primates (Mac 1, 2, and 9) [4]. This is consistent with a vector copy number ranging from 9 to 11.8 vector genomes per diploid genome (vg/dge) at 20 months pi at ≈1 cm from the injection site in the tibialis anterior muscle. However, these primates also had a substantial number of vector copies in the liver, from 0.2 to 3.4 vg/dge at 31 months pi. Since LEA29Y is a secreted protein, we could not correlate the methylation status of rAAV genomes with the local transgene expression by assessing the protein level. Thus, we developed a quantitative RT-PCR (Q-RT-PCR) assay to determine the vector mRNA copy number in each transduced tissue (skeletal muscle and liver). Vector mRNA relative quantities (RQ) were determined by normalizing Ct values to the HPRT internal reference that is similarly expressed in different rhesus monkey tissues [36]. RQ was then reported as a ratio to the vector genome copy number (i.e. RQ/vg) for each sample. Total RNA was extracted from the 5 injected primates and Q-RT-PCR quantification gave values ranging from 0.02 to 1.98 RQ/vg in the liver and from 104.51 to 2355.48 RQ/vg in the muscle samples more than 1 year after injection. Results obtained for Mac 9 are represented on Figure 5 where the RQ/vg ratio was 1480 fold higher in the muscle than in the liver at the later time point (37 months pi). As controls, Q-RT-PCR analysis was performed on macaque Myosin-4 (MYH4) and Albumin (ALB1) mRNA samples obtained from Mac 9. We confirmed that these genes were specifically transcribed in skeletal muscle or liver, respectively, with the MYH4 mRNA undetectable in the liver and the RQ values ranged from 13.4 to 191.4 per copy of MYH4 gene in the muscle. Inversely, RQ per ALB1 copy was equal to 0.23+/−0.38 on average in Mac 9 muscle and ranged from 1293.3 to 1940.8 in the liver. In conclusion, the rAAV delivered RSVp was much more efficient for driving transgene expression in NHP muscle compared to the liver. Considering that most of the vector genomes were detected in the muscle and the liver after IM administration of rAAV vectors (Le Guiner *et al.*, unpublished data), the majority of the LEA29Y protein expression derives from the injected skeletal muscle.

Since no DNA methylation was found in the RSVp either in the skeletal muscle or in the liver, our data demonstrate that the partial decrease of transgene expression during Phase 2 is not due to DNA methylation and suggests the involvement of other regulatory mechanisms.

## Discussion

In the study presented here, we assessed the involvement of DNA methylation in the partial repression of transgene expression after rAAV vector administration in NHP skeletal muscle. In this clinically relevant model, low-throughput pyrosequencing (LTS) [26] and high-throughput 454 sequencing (HTS) [39] demonstrated that the RSVp is not subject to *de novo* CpG methylation at early or late time points in NHP muscle and liver. When the population or individual rAAV molecules are analyzed by pyrosequencing, no methylation was seen. Thus, the reduction in protein levels observed in Phase 2 is not due to the methylation of a portion of the rAAV genome population. Other mechanisms are likely involved such as the loss of some rAAV forms or deletions/rearrangements that are known to occur during rAAV genome circularization and concatemerization. Sun *et al.* recently suggested that recombination of ITR and adjacent regions after transduction of NHP liver may impact the integrity of the transgene cassette, particularly in the promoter due to its close proximity to the ITR [10].

Pyrosequencing is a recent technology that enables the evaluation of the DNA methylation percentage for each CpG position in a population of molecules converted by sodium bisulfite and amplified using PCR [26], [27]. It circumvents the poor sensitivity and the biases associated with classical sub-cloning and Sanger sequencing approaches [25]. In our assay, the *in vitro* methylated control appeared to be 96.25% methylated overall which was satisfactory considering the under-estimation of high levels of methylation using pyrosequencing technology [25], and incomplete enzymatic methylation by M.Sss1 methyltransferase [40]. We demonstrated that no methylation was detectable in the promoter region of the packaged rAAV genomes. Since CpGs of rAAV genomes are unmethylated in virions, this could increase the susceptibility to TLR9 recognition in the endosomes and promote AAV-targeted adaptive immune responses [24], [41]. Indeed, Zhu *et al.* demonstrated that the TLR9-MyD88 pathway was involved in the activation of CD8+ T cell responses to both the transgene product and the rAAV2 capsid, leading to loss of transgene expression [42].

In order to evaluate the potential *de novo* methylation *in vivo*, the percentage of DNA methylation was measured along the RSVp from 1 month to 37 months after rAAV2/1 administration in NHP. Some liver samples (indicated as “na” on **Fig. 3b**) could not be analyzed by LTS probably because mutations/rearrangements could have occurred in the RSVp sequence during circularization and concatemerization of rAAV genomes [10]. By providing resolution of individual CpG, LTS allowed visualization of methylation variation between CpG positions in the population. The Pyro1 region was found in the first instance slightly methylated for most of the samples, while Pyro2 remained unmethylated. Interestingly, most transcription factor (TF) binding sites are located in the Pyro2 region (Fig. 1c); and this region has been described as indispensable for RSVp-driven transcription [43]. In order to confirm these results, we chose to perform high-throughput sequencing on these samples using 454 HTS since this approach allows the simultaneous analysis of 400,000 individual molecules on a single picotiter plate with a single-read sequence error of less than 0.5% [39], [28]. Moreover, HTS provided additional information concerning the distribution of the CpG methylation in the population of rAAV genomes. Contrary to the LTS results, no significant DNA methylation of the RSVp was detected by HTS. Since HTS generates individual clonal sequence reads, incomplete bisulfite converted sequences that can cause rare false positives could be identified based upon assessment of non-CpG cytosines. These false positives sequence reads appeared to be shorter than the expected sequence and thus were removed using optimized cut-offs applied during bioinformatic analysis. This additional selective filter could explain the lower threshold observed in HTS compared to LTS (1.87% and 0.60%, respectively) as well as the higher reproducibility. Overall, the methylation levels obtained by LTS and HTS were similar considering the described technical variability, due in part to errors known to occur in homopolymeric nucleotides stretches [28].

One cannot exclude that the RSV promoter *per se* is responsible for the resistance of the rAAV DNA to methylation since it is known as a strong constitutive promoter [44]. However, several *in vitro* studies demonstrated that the partial or complete CpG methylation of the RSVp prior to transfection could severely alter the transgene transcription level [32], [33], [45], [46]. The plasmid used in these studies contained an EBNA1/OriP sequence that conferred plasmid chromosome attachment and maintenance during cell division [12]. In fact, the RSVp was found to be susceptible to *de novo* methylation only when integrated, in clones generated by plasmid transfection with antibiotic selection [47]. Since rAAV vectors are maintained nearly exclusively as episomes in skeletal muscle [4], [48] and liver [10], [11], silencing due to integration into heterochromatin should not contribute significantly to repressing rAAV-mediated transgene expression. Thus, with the exception of a single sample (Mac 10 liver at 1 month pi), the rAAV integration frequency was likely too low in our primate samples to detect DNA methylation associated with such silencing. Indeed, we failed to detect any integration event by LAM-PCR (linear amplification-mediated PCR) in Mac 1, Mac 2 and Mac 9 injected muscles [4]. Furthermore, it has been recently demonstrated that the silencing of transgene expression in mouse liver by plasmid bacterial backbone DNA was consistent with the histone modifications profile but was independent of CpG methylation [22], [23]. Similarly, naked plasmid was recently shown to be unmethylated along the hAAT promoter after hydrodynamic liver-directed delivery in mice, which was consistent with a stable FIX transgene expression for 3 months [49]. We hypothesized that, like plasmid DNA, the episomal status of rAAV could result in the absence of *de novo* DNA methylation in these quiescent tissues.

Finally, we demonstrated by Q-RT-PCR that the RSVp was highly active in NHP skeletal muscle but restricted in the liver after ssAAV2/1 vector administration. This was consistent with Salva *et al.* data obtained in mice after single-stranded AAV6 injection [50]. They showed that the alkaline phosphatase (AP) reporter protein was poorly expressed in the liver while it was highly expressed in many other organs, including the skeletal muscle, 1 month after rAAV-RSV-AP IV injection. The constitutive CMV promoter was also shown to drive a 10-fold greater AP activity than the RSVp in the liver [50]. Similar, we observed a much higher RSV-driven transgene expression in the skeletal muscle of mice that were injected IM with a ssAAV2/8-RSV-GFP vector, compared to the liver (data not shown). In conclusion, the RSVp used in a context of ssAAV is a suitable promoter to drive expression in skeletal muscle, but a very weak promoter in NHP and murine liver.

Since this study was carried out in normal quiescent tissues, DNA methylation inheritance mechanisms that occur during DNA replication and involve the DNA methyltransferase 1 (Dnmt1) are not expected to play a role. However, other DNA methyltransferases, Dnmt3a and 3b, also called *de novo* DNA methyltransferases, are expressed in the skeletal muscle and the liver and are capable of methylating single- or double-stranded unmethylated DNA [51]. Whereas global differential methylation is a well-described mechanism occurring during embryonic development [52], the extent and the role of *de novo* methylation in adult quiescent tissues remain unclear. Thus, it could be interesting to monitor DNA methylation and, more generally, additional epigenetic modifications in a regenerative cellular context such as dystrophic muscle, to correlate the potential impact on vector-derived transgene expression.

## Supporting Information

Table S1 **PCR primers used for high-throughput 454 sequencing analysis.** Underlined sequences correspond to 454-bead attachment sequence (A or B). Sequences in bold correspond to the tags or Multiplex identifier (MID) sequences and in normal font to the target-specific sequences.
(TIF)
