# Supplementary figures and images for: Adeno-Associated Viral Vector-Mediated Transgene Expression Is Independent of DNA Methylation in Primate Liver and Skeletal Muscle
Source: PLoS One. 2011 Jun 8;6(6):e20881. doi: 10.1371/journal.pone.0020881 (PMC3110818; doi:10.1371/journal.pone.0020881)

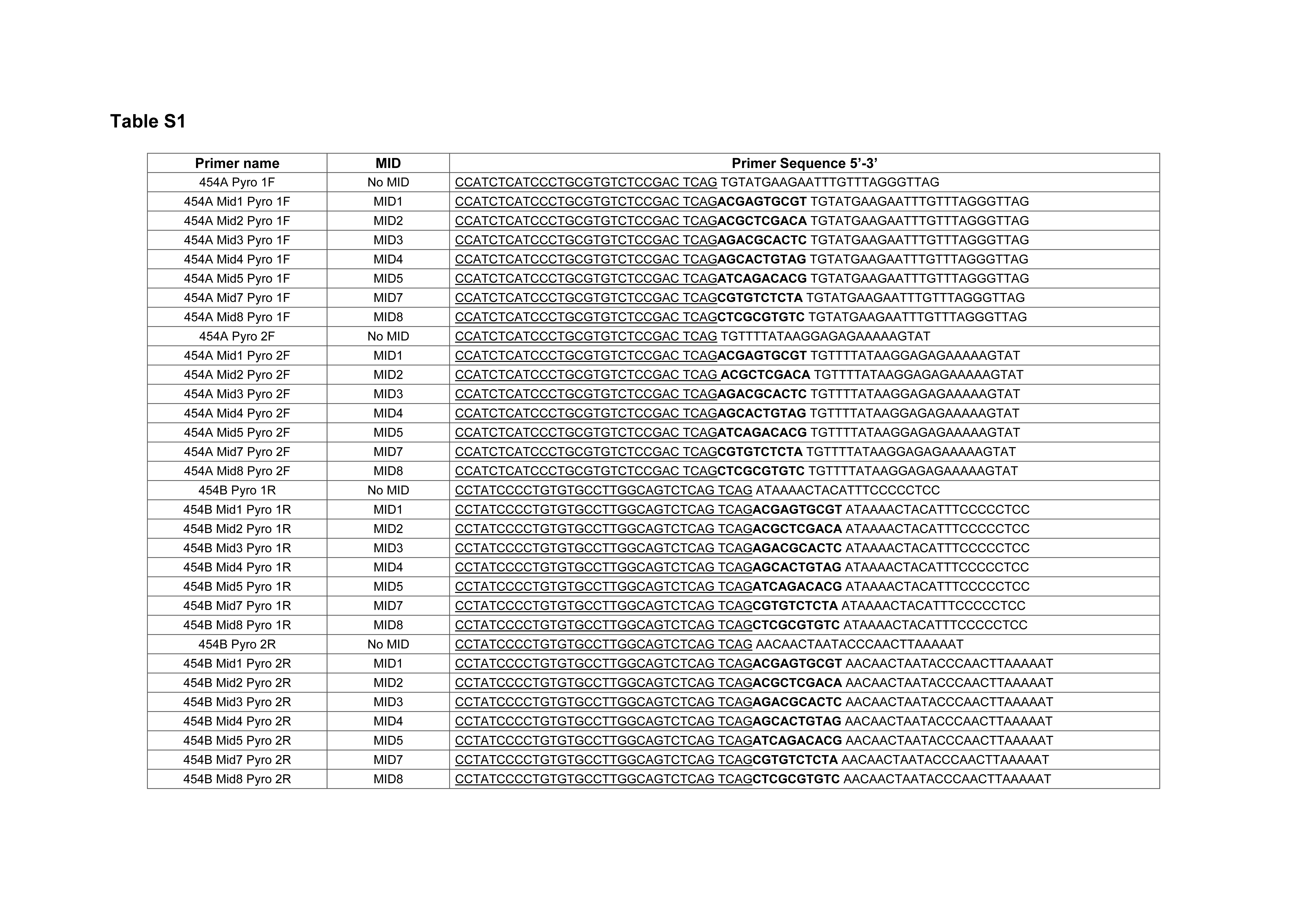

Supplement: Table S1 — PCR primers used for high-throughput 454 sequencing analysis. Underlined sequences correspond to 454-bead attachment sequence (A or B). Sequences in bold correspond to the tags or Multiplex identifier (MID) sequences and in normal font to the target-specific sequences. (TIF) [file pone.0020881.s001.tif]
